# Neuropeptide Y Enhances Olfactory Mucosa Responses to Odorant in Hungry Rats

**DOI:** 10.1371/journal.pone.0045266

**Published:** 2012-09-14

**Authors:** Julia Negroni, Nicolas Meunier, Régine Monnerie, Roland Salesse, Christine Baly, Monique Caillol, Patrice Congar

**Affiliations:** 1 INRA, UR1197 Neurobiologie de l′Olfaction et Modélisation en Imagerie, Jouy-en-Josas, France; 2 IFR144, NeuroSud Paris, Gif-Sur-Yvette, France; 3 Université de Versailles Saint-Quentin en Yvelines, Versailles, France; Duke University, United States of America

## Abstract

Neuropeptide Y (NPY) plays an important role in regulating appetite and hunger in vertebrates. In the hypothalamus, NPY stimulates food intake under the control of the nutritional status. Previous studies have shown the presence of NPY and receptors in rodent olfactory system, and suggested a neuroproliferative role. Interestingly, NPY was also shown to directly modulate olfactory responses evoked by a food-related odorant in hungry axolotls. We have recently demonstrated that another nutritional cue, insulin, modulates the odorant responses of the rat olfactory mucosa (OM). Therefore, the aim of the present study was to investigate the potential effect of NPY on rat OM responses to odorants, in relation to the animal's nutritional state. We measured the potential NPY modulation of OM responses to odorant, using electro-olfactogram (EOG) recordings, in fed and fasted adult rats. NPY application significantly and transiently increased EOG amplitudes in fasted but not in fed rats. The effects of specific NPY-receptor agonists were similarly quantified, showing that NPY operated mainly through Y1 receptors. These receptors appeared as heterogeneously expressed by olfactory neurons in the OM, and western blot analysis showed that they were overexpressed in fasted rats. These data provide the first evidence that NPY modulates the initial events of odorant detection in the rat OM. Because this modulation depends on the nutritional status of the animal, and is ascribed to NPY, the most potent orexigenic peptide in the central nervous system, it evidences a strong supplementary physiological link between olfaction and nutritional processes.

## Introduction

Most animals, including humans, rely on their sense of smell for food seeking, food choice and the appreciation of food palatability [1], [2]. Moreover, olfactory neural processing is closely linked to the physiological and nutritional status of an organism: the olfactory system is more active [3], [4], [5] and its sensitivity [6] is increased under starvation, whereas both activity and acuity are reduced after satiation [3], [4], [5], [6]. While this relationship has been known for several decades, the signaling systems and the mechanisms underlying the modifications of the olfactory activity induced by the nutritional state were only recently explored. There is a growing body of evidence suggesting that several nutritional and metabolic cues, including orexins, leptin and insulin, modulate the peripheral steps of odor detection in rodents [5], [7], [8], [9], [10], [11], [12], [13], [14], [15].

NPY is one of the most abundant [16] and the most potent orexigenic peptide [17], [18], [19] in the central nervous system where it is widely expressed [20], [21], [22]. NPY acts through a family of at least five G-protein-coupled receptors, which are broadly expressed in the developing and adult brain [23], [24], [25], [26], [27]. Among numerous physiological functions (for review see [28], [29]), NPY plays a pivotal role in the control of food intake, mainly through central hypothalamic sites, in numerous animal species [17], [30], [31], [32], [33], [34], [35], [36].

Recent studies have shown the presence of NPY in both the developing and the adult OM of rodents, and suggested its implication as a neuroproliferative factor [37], [38], [39]. NPY is primarily expressed within a subset of developing embryonic neurons and basal cells, whereas in the postnatal OM it is mostly expressed in OSNs [40], sustentacular cells [37], [39], [41], microvillar cells [42] and olfactory ensheathing cells [43]. In both cases it is thought to act through the Y_1_ receptor (Y_1_R) to stimulate proliferation of olfactory sensory neuron (OSN) progenitors [37], [38], [40], [44]. The synthesis and the release of NPY are locally promoted by ATP, through the activation of P2Y purinergic receptors, in both neonatal and adult OM [39], [41]. Finally, blockade of NPY effects by the selective knockout of Y_1_R produces a significant reduction in OSN precursor proliferation, which was hypothesize to result in a moderate impairment of olfactory function [40]. Taken together, these data clearly show that NPY plays an important role in the neonatal proliferation and the renewal of the adult olfactory system.

NPY was also shown to affect the odorant detection in amphibians: it directly modulates olfactory responses evoked by a food-related odorant in hungry axolotls [45]. However, the possibility of an acute neuromodulatory role of NPY has never been explored in the olfactory system of mammals.

**Figure 1:**
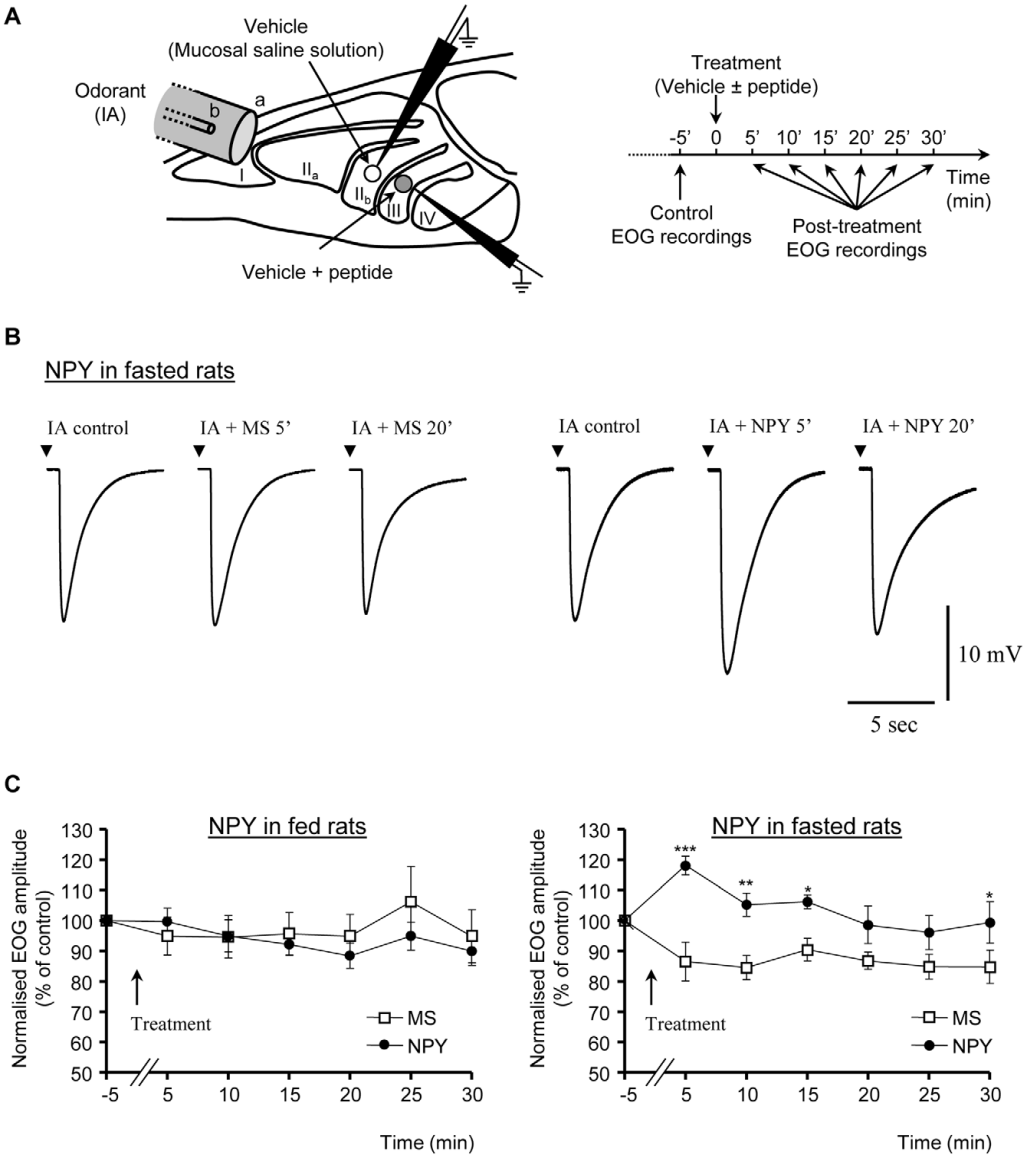
NPY enhances olfactory responses in fasted rats. (A) Schematic diagram of the experimental preparation. Turbinates were exposed in rat hemi-heads; odorant (IA, isoamyl acetate) was delivered rostrally through a Pasteur pipette (b) enclosed in a glass tube (a). The recording electrodes are represented on turbinates II_b_ and III. The time course of recordings and NPY (or its agonists) treatment are described on the right. (B) Typical EOG responses to IA in an overnight fasted rat, before and after treatment with mucosal solution (MS) alone (*left*) or containing 1 µM of NPY (*right*). (C) Effects of MS or MS + NPY (1 µM) treatments on the time course of EOG amplitudes in response to IA in fed (B; n = 10) and overnight fasted (C; n = 10) rats. Mean ± SEM values are expressed as a percent of the baseline responses (prior to treatment). (*P<0.05; **P<0.01; ***P<0.001).

In the present study, EOG recording techniques and pharmacological approaches were used to examine the possibility that NPY could acutely modulate the olfactory responses in rats. We found that NPY increases the olfactory responses in the OM of fasted but not of fed rats, mainly through Y_1_ receptors. Morevover, immunohistological markers and western blot analysis allowed us to show that Y_1_R are heterogeneously expressed by OSNs and overexpressed in the OM of fasted rats. Our results demonstrate that olfactory sensory neurons from hungry animals are more responsive to NPY, thereby providing a supplementary functional basis for the modulation of olfaction by the nutritional state.

**Figure 2:**
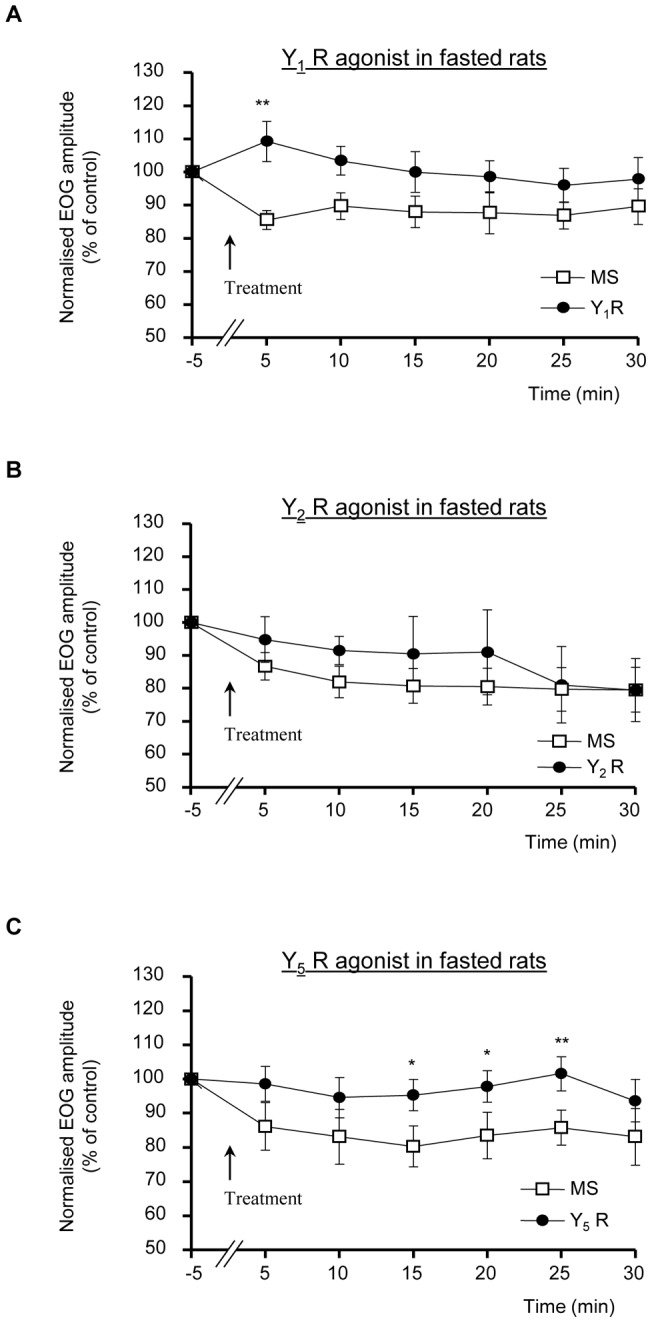
NPY effect is mimicked by Y_1_ receptor agonist. Effects of MS or MS + Y_1_R agonist (A), Y_2_R agonist (B) or Y_5_R agonist (C) treatments (1 µM) on the time course of EOG amplitudes in response to IA in fasted rats. Mean ± SEM values (n = 10, 5 and 10 rats, respectively for Y_1_, Y_2_ and Y_5_ agonist) are expressed as % of control EOG recordings prior to treatment. (*P<0.05; **P<0.01).

## Methods

### Animals

Experiments were carried out on young adult (2 months) male Wistar rats born in our local animal care facilities (Unité Expérimentale d'Infectiologie Expérimentale des Rongeurs et Poissons (UEIERP), Jouy-en-Josas, France). All animal experiments were conducted in accordance with the European Communities Council Directive of November 24, 1986 (86/609/EEC). P.C. and C.B. hold the Individual Authorization for Performing Experiments in Animals, including the animals experiments conducted in the present study, provided by Préfecture des Yvelines (France), according to French and European laws (agreements #78–154 and #78–65). All surgery was performed under sodium pentobarbital anesthesia, and all efforts were made to minimize the number and suffering of used rats.

They were kept under controlled conditions of light (12 h light and 12 h dark, lights on at 07∶00) and temperature (22°C) with free access to pellet food (M25, Dietex, Saint-Gratien, France) and tap water. To determine the effect of fasting, food but not water was withheld for 14 h before tests. For all experiments, rats were killed at the beginning of the light phase (09∶00–10∶00) by decapitation.

### Electro-olfactogram (EOG) recording

EOG recordings were made from the OM in an opened nasal cavity configuration, on rat hemi-heads. Immediately after decapitation, the head was cut longitudinally and the nasal septum was removed from one hemi-head to expose the endoturbinates from which EOG recordings were performed at room temperature (Fig. 1A). The OM tissue was simultaneously dissected from the contralateral hemi-head and stored at −80°C for subsequent western blot analysis (see below).

The EOG recordings were done using an experimental design described earlier [14]. The hemi-head was placed in a recording chamber on a custom-designed platform (Siskiyou, Inc., Grants Pass, OR, USA) of an upright Olympus BX51WI microscope (Olympus, Rungis, FRANCE) equipped with a low magnification objective (4x) and MPC-325 motorized micromanipulators (Sutter Instruments, Novato, CA, USA). The odor stimulation device was modified from Scott and Brierley [46]. The hemi-head was kept under a constant flow of humidified filtered air (∼1200 ml/min) delivered close to the endoturbinates through a 9 mm glass tube (Fig. 1A (a)). This tube was positioned 2 cm from the epithelial surface and was centered on the recorded endoturbinates. Odor stimulations were performed by blowing air puffs (100 ms, 300 ml/min) through an exchangeable Pasteur pipette (Fig. 1A (b)) enclosed in the glass tube and containing a filter paper impregnated with 20 µl of the odorant, isoamyl acetate (IA; Sigma Aldrich (Saint-Quentin Fallavier, France)), diluted 1: 100 in DMSO. To prevent variable accumulations of the odorant in the pipette, an air flush was applied to the Pasteur pipette before it was placed in the glass tube and before each subsequent odorant application.

EOG voltage signals were recorded using an Multiclamp 700B patch-clamp amplifier (Axon Instruments, Molecular Devices, Union City, CA, USA) used in a DC current-clamp configuration (I = 0), low-pass bessel filtered at 1 KHz and digitized at a rate of 2 kHz using an Digidata 1322a A/D converter (Axon Instruments, Molecular Devices, Union City, CA, USA) interfaced to a Pentium PC and Pclamp 9.2 software (Axon Instruments). A reference Ag/AgCl electrode was placed on the frontal bone overlaying the olfactory bulb. Recordings were made with glass micropipettes of 4–5MΩ filled with a mucosal saline (MS) solution (45 mM KCl, 20 mM KC_2_H_3_O_2_, 55 mM NaCH_3_SO_4_, 1 mM MgSO_4_, 5 mM CaCl_2_, 10 mM HEPES, 11 mM glucose, 50 mM mannitol, pH 7.4, 350 mOsm adjusted with mannitol). The composition of this solution was chosen to match the composition of mucus as closely as possible, according to previous studies [47]. Simultaneous EOG recordings of the responses evoked by isoamyl acetate, were recorded from the neighboring endoturbinates II_b_ and III. The recording electrodes were placed in the center of these endoturbinates (Fig. 1A). This position gave robust, reproducible and long-lasting EOG recordings ranging from 10 to 30 mV in response to IA. Odorant-free air stimulation, or stimulation with DMSO alone, always produced signals of less than 1 mV amplitude.

EOG responses were recorded until their amplitudes stabilized, (i.e. at least 3 successive responses displaying the same amplitude), and the last response was taken as the reference. One endoturbinate was treated by a local application of the mucosal saline solution (MS), while the other endoturbinate received the same MS containing the tested peptide (1 µM NPY or NPY-receptor agonist). Droplets of approximately 1 µl of the solution were delivered by capillarity onto the recorded area of endoturbinates II_b_ or III (respectively white and grey circles in Fig. 1A) using glass micropipettes (∼5 µm in diameter). One droplet covered about 1–2 mm diameter of the olfactory mucosa on a given endoturbinate, as checked under the microscope. MS and peptide-containing-MS treatments were systematically shifted between the two endoturbinates (II_b_ and III) from one rat hemi-head to the other. Responses to IA were then recorded on each endoturbinate every 5 min, for 30 min following local treatment (Fig. 1A). The effect of NPY was compared between fed (n = 10 rats) and fasted (n = 10 rats) rat hemi-heads, whereas the effects of NPY-receptor agonists were studied in overnight-fasted rat hemi-heads (n = 10, 5 and 10 rats, respectively for Y_1_, Y_2_ and Y_5_ receptor agonist). Analysis were performed off-line using Clampfit 9.2 (Axon Instruments). Peak amplitude, rise time (from 10% to 90%), fast (from 100% to 50%) and slow (from 40% to 10%) decay slopes of EOG responses were measured. Since the EOG response decay kinetics highly correlate with the amplitude, the two decay slopes were normalized to the corresponding response peak amplitude prior to statistical analysis. The amplitude and kinetics of the olfactory signals recorded following the local peptide applications were normalized to those of the control responses prior to this treatment.

### Y_1_R Western blotting

In order to quantify Y_1_R protein, the OM tissue dissected from fed (n = 5) and fasted (n = 11) rat hemi-heads was homogenized in a volume of extraction buffer (25% glycerol, 0.42 M NaCl, 1.5 mM MgCl_2_, 0.2 M EDTA, 20 mM Hepes; pH = 7.5) containing 1∶100 of phenyl-methylsulphonyl fluoride (PMSF), 1% Nonidet-P-40 and 1∶25 of a cocktail of anti-proteases (Complete; Roche Diagnostics, Meylan, France). These tissue extracts were then centrifuged at 16000 g for 20 min, the supernatants were collected and protein levels quantified using BCA protein assay (Pierce, Perbio Science, Brébières, France). To test for the presence of Y_1_R, we performed an 8% SDS-PAGE analysis of boiled aliquots (75 µg of total proteins) from each extracts. Following electrotransfer, membranes were blocked with 4.5% nonfat milk and then incubated overnight at 4°C with a rabbit polyclonal Y_1_R antibody (ab 73897; 1∶500; Abcam, Cambridge, UK) or a mouse monoclonal β-actin antibody (chosen as the reference protein) (A5441; 1∶3000; Sigma Aldrich, Saint-Quentin Fallavier, France). After extensive washing in PBS-0.5% milk (4×15 min each), the membranes were incubated with horseradish peroxidase-conjugated anti-rabbit secondary antibody (1∶5000; Sigma Aldrich, Saint-Quentin Fallavier, France). The targeted proteins were detected using an ECL western blotting detection kit (Amersham Biosciences, Orsay, France). The integrated density of protein level of each band on the blots was determined by densitometry using ImageJ software (free Java port of NIH image, http://rsb.info.go/ij/).

### Immunohistochemistry (IHC)

Fed (n = 4) and fasted (n = 5) rats were deeply anaesthetized with sodium pentobarbital (CEAV, Libourne, France; 100 mg/kg) and perfused transcardially with 200 ml saline and then with 300 ml of a freshly prepared fixative solution of 4% paraformaldehyde in 0.1 M phosphate-buffered saline (PBS). The skull was opened and the brain was carefully dissected. The bones of the skull were discarded from the lateral and dorsal walls of the nose and the olfactory mucosa (septum and turbinates) with the olfactory bulb were removed as a block. Tissues were post-fixed in the same fixative for 3 h at room temperature, cryoprotected with saccharose (30%) and cut using a cryostat into 14 μm thick serial horizontal sections. The slide-mounted sections were stored at −80°C until use. Upon request, tissue sections were rehydrated with three PBS washes. In order to block endogen peroxidase, the sections were treated at room temperature with 1% H_2_O_2_ for 30 min. The non-specific binding sites were blocked for 30 min at room temperature with a non-immune serum issued from the host species of the secondary antibody (usually 1∶10 normal goat serum in PBS containing 0.25% Triton X-100, and 2% bovine serum albumin). Sections were then incubated with the primary antibody in Triton (0.05%) – BSA (0.2%) –1∶10 normal goat serum – PBS for 48–72 h at 4°C. A polyclonal antibody raised in rabbit was used to localize Y_1_R in the OM (ab 73897, 1∶250, Abcam, Cambridge, UK). A monoclonal antibody raised in mouse was used to localize β-tubulin isotype III (clone SDL.3D10, 1∶200, Sigma Aldrich, Saint-Quentin Fallavier, France). Controls were performed using incubation with non-immune serum in place of the primary antibody; no immunostaining was observed when the primary antibodies were omitted. After washes in PBS, labeling was visualized using secondary antibodies conjugated either with fluorochrome (Alexa-Fluor-488- and Alexa-Fluor-546-conjugated secondary antibodies, raised in goat, Molecular Probes, Invitrogen, Cergy Pontoise, France, 1: 1000, 1 h at room temperature) or biotin revealed with a biotin–avidin–peroxidase kit (Vectastain ABC Kit, Vector Laboratories, AbCys, Paris) using diaminobenzidine (DAB) as a chromogen in presence of H_2_O_2_ and nickel ammonium sulfate (DAB substrate kit for peroxydase, Vector Laboratories, AbCys, Paris). For immunofluorescence detection, preparations were washed in PBS and mounted in DAPI-containing Vectashield (AbCys, Paris, France). For DAB detection, sections were air dried, dehydrated in ethanol, cleared in xylene and mounted in DePeX (GURR, Labonord, Villeneuve d'Ascq, France).

### Images processing

IHC images were acquired either on a DMBR Leica microscope equipped with an Olympus DP-50 CCD camera using Cell^F^ software (Olympus Soft Imaging Solutions GmbH, OSIS, Münster, Germany), or on an AxioObserver.Z1 microscope equipped with a structured illumination system (Zeiss Apotome, Oberkochen, Germany) using Axio Vision 4.8 (Zeiss). Apotome observations were made at the Mima2 facilities in Jouy-en-Josas (France). Images were cropped, resized, rotated, merged, and adjusted for contrast and brightness, for presentation purposes, using ImageJ, but their content was not altered in any case.

### Chemicals

All chemicals were dissolved in distilled water or DMSO when required. Concentrated stock solutions were kept frozen at −20°C, and diluted extemporaneously to their final concentrations in the appropriate saline solutions. Neuropeptide Y (Human, Rat) Trifluoracetate Salt was purchased from Bachem (Weil am Rhein, Germany). Selective agonists of Y_1_ ([Phe7, Pro34] pNPY), Y_2_ (Ahx [5]–[24] pNPY) and Y_5_ ([Ala31, Aib32] pNPY) NPY receptors subtypes were generously donated by Prof. Dr. Beck-Sickinger from the Institut of Biochemistry in Germany (Leipzig University). A commercial Y_1_-receptor agonist ([Leu 31, Pro 34] Neuropeptide Y; NeoMPS, Strasbourg, France) was alternately used to confirm the results obtained with EOG recordings. The rabbit polyclonal anti-Y_1_R antibody (ab73897) and the Y_1_R immunogen peptide (ab82262) were both purchased from Abcam (Cambridge, UK). All other chemicals were purchased from Sigma Aldrich (Saint-Quentin Fallavier, France).

### Statistical analysis

Group data presented in the text and figures are expressed as mean ± standard error of the mean (SEM). For EOG data analysis, we first used a 2-way analysis of variance (ANOVA) to determine the overall significance of the effects of time, MS, NPY or agonist treatments. When the ANOVA indicated a significant effect, post hoc Bonferroni multiple comparison of mean tests was used to determine individual differences between responses at each time considered, and differences over time for each treatment. For western blot analysis, statistical comparisons were performed using the Welch's *t*-test (intended for use with two samples having unequal variances). A probability value of p<0.05 was used as an indication of significant differences (p<0.05 (*); p<0.01 (**); p<0.001 (***)).

## Results

### NPY enhances EOG responses in the OM of fasted rats

The potential neuromodulatory role of NPY in the OM was studied using EOG recordings of the responses evoked by an odorant, isoamyl acetate (IA), both in fed (n = 10) and overnight-fasted (n = 10) animals. We compared the effects of NPY and vehicle (mucosal saline solution (MS)) applications on the olfactory responses in each recorded rat hemi-head. Simultaneous EOG recordings of the responses evoked by IA were recorded from the neighboring endoturbinates IIb and III (Fig. 1A). One endoturbinate was treated by a local application of MS (white circle, Fig. 1A), while the other endoturbinate received the same MS containing the tested peptide (1 µM NPY or NPY-receptor agonist; grey circle, Fig. 1A). MS and peptide-containing-MS treatments were systematically shifted between the two endoturbinates (II_b_ and III) from one hemi-head to the other, in each condition (fed/fasted). Local treatment of the OM with MS did not significantly change the amplitude and the kinetics of IA-induced olfactory responses (Fig. 1B–C) recorded up to 30 min post-treatment. In both fed and fasted animals, the EOG responses amplitudes displayed slight and gradual decrease over time (about 15% within 30 to 45 min of recording), as previously reported [48]. In both fed and fasted animals, two-ways ANOVA analysis showed no significant effect of MS treatment, and no difference between the time-course of the EOG amplitudes observed in both conditions. In fed animals, the local treatment with NPY (1 µM) did not significantly change this slow temporal waning (n = 10; fig. 1B). In contrast, in fasted animals, local application of NPY raised the olfactory responses (n = 10; Fig. 1C). The two-ways ANOVA analysis revealed a significant effect of NPY (p<0.01), significantly different time-courses of the EOG amplitudes between MS and NPY treatments (p<0.01), and a significant treatment x time-course interaction (p<0.01). NPY induced a transient increase of the amplitude of IA-evoked EOG responses, reaching a maximum, around 30% above the corresponding MS-treated EOG amplitudes, 5 min after the application (118.1±3.1% and 86.6±6.4% of control EOG amplitude, respectively in NPY- and MS-treated groups; n = 10; p<0.01), and lasting for up to 15 min. The EOG-amplitudes then, followed the progressive decay observed in the MS treated animals, despite a small but significant rebound at 30 min (n = 10, fig. 1C). In contrast to its potentiating effect on the amplitude, NPY did not modify the kinetics of IA-evoked responses in fasted rats (not shown).

**Figure 3:**
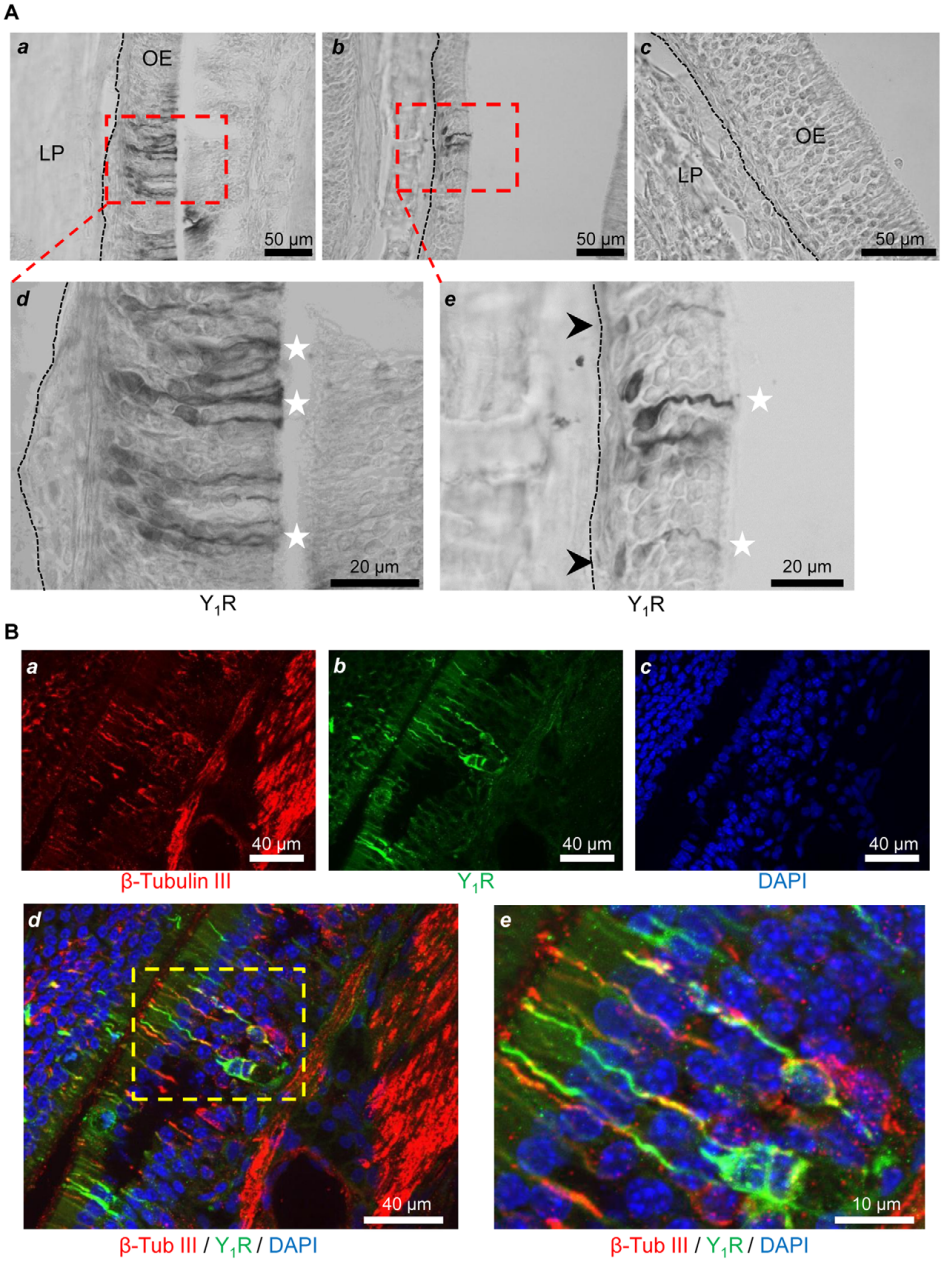
Y_1_ receptor is prominently located in OSNs. (A) Bright field microscopy images of OM sections from endoturbinates of an overnight fasted rat showing the neuronal localization of Y_1_R revealed with DAB (light microscope) at medium (40x) (a and b) and high (100x) (d and e) magnification. Stronger signal was detected in the lower half of the epithelium, corresponding to olfactory sensory neurons (OSNs) (white stars), and in few more basally situated cells (e, black arrowheads). No labeling was observed in control sections incubated with non-immune rabbit serum (c). The dashed line indicates the limit between the olfactory epithelium (OE) and the lamina propria (LP). (B) Immunofluorescence images of OM sections from endoturbinates of an overnight fasted rat showing the neuronal localization of Y_1_R (B-a, green) with OSN marker, β tubulin III (B-b, red) by double labeling. Cell nuclei were counterstained with DAPI (B-c, blue). Y_1_R expression was observed in β tubulin III-positive OSNs (B-d), more clearly seen at higher magnification (B–e).

We next aimed to determine the subtype of NPY receptor implicated in this positive modulation of the olfactory responses in fasted rats. While Y_1_, Y_2_ and Y_5_ are the most prevalent NPY receptors in the mammalian brain, Y_1_ receptors mediate most of the previously reported actions of NPY in the olfactory system. We thus compared the effects of selective agonists of Y_1_, Y_2_ and Y_5_ receptors [49], [50] to the control MS application on the EOG responses recorded in overnight-fasted rats (Fig. 2). As with NPY, the selective application of Y_1_ agonist [Phe7, Pro34] pNPY (1 µM; [51]) induced a significant transient increase of the amplitude of IA-induced EOG in overnight-fasted rats, reaching a maximum, around 25% above the corresponding MS-treated EOG amplitudes, 5 min after the application (108.7±6.3% and 85.5±2.8% of control EOG amplitude, respectively in Y_1_ agonist- and MS-treated endoturbinates; n = 10; fig. 2A; p<0.01). Moreover, this Y_1_-mediated increase appeared to be slightly more transient than the NPY effect, as the two-ways ANOVA analysis revealed a significant effect of Y_1_ agonist (p<0.05), but no difference in the time-course of the EOG amplitudes between MS and Y1 agonist treatments (and no treatment x time-course interaction). In contrast, local treatment with the selective Y_2_ receptor agonist Ahx [5]–[24] pNPY (1 µM; [52]) did not change the amplitude or time-course of the olfactory response amplitudes in fasted rats (n = 5, fig. 2B). Finally, local treatment with the selective Y_5_ agonist [Ala31, Aib32] pNPY (1 µM; [53]) induced a slight and delayed increase of the amplitude of IA-induced EOG in overnight-fasted rats, slowly reaching a maximum, around 15% above the corresponding MS-treated EOG amplitudes, 25 min after the application (101.6±5.0% and 85.7±5.1% of control EOG amplitude, respectively in Y5 agonist- and MS-treated endoturbinates; n = 10; fig. 2C; p<0.01). The two ways ANOVA analysis revealed no difference in the time-course of the EOG amplitudes between MS and Y5 agonist treatments (and no treatment x time-course interaction). However, as expected from the NPY effect, none of the three selective agonists tested changed the kinetics of the IA-induced EOG responses (not shown).

**Figure 4:**
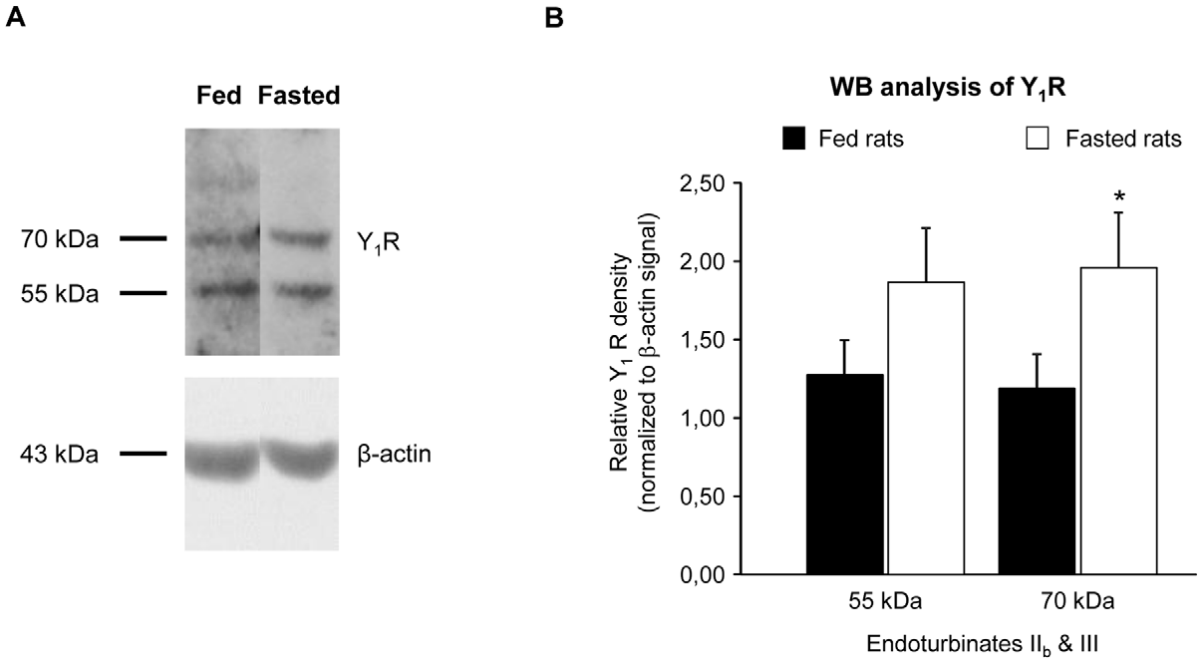
Y_1_ receptor expression is up-regulated in the OM of fasted rats. (A) Immunoblotting for Y_1_R in OM extracts from fed and fasted rats reveals two main bands (55 and 70 kDa) on the western blot membranes. (B) Comparison of Y_1_R protein expression level in the OM (endoturbinates II_b_ and III) of fed (n = 5) and fasted (n = 11) rats. The integrated density of each band on the western blots was determined by densitometry using ImageJ software, and normalized to β-actin signal. Each sample was measured on two independent gels. Results from both blots were pooled and are expressed as mean ± SEM; significant difference is denoted by * P<0.05 (Welch's t-test).

Thus, on the basis of these electrophysiological data, NPY application significantly and transiently increased EOG amplitudes in fasted but not in fed rats. This increase of olfactory response amplitudes appeared to mainly imply a prominent fast-developing Y1-mediated effect, followed by a slight slowly-developing Y5-mediated component.

### NPY-Y_1_ receptors are localized in OSNs

Since the most prominent part of the NPY positive modulation was mediated via the activation of the Y_1_ type receptor, we used immunohistochemical methods to identify the different cell types expressing Y_1_R in the OM of fed (n = 4) and fasted rats (n = 5). We found Y_1_R heterogeneously expressed in the OM. Most of the labeled cells were morphologically similar to olfactory sensory neurons (OSNs), with cell somas mostly located in the lower half of the epithelium, grouped in patches of various number of Y_1_R immunoreactive cells (Fig. 3A–B). In addition to these cells, few labeled cells were localized close to the lamina propria, in the basal cell layer of the OM (Fig. 3B–b). When the Y1R primary antibody was replaced by non-immune rabbit serum, no immunostaining was observed (Fig. 3A–c). To confirm the Y_1_R expression in OSNs, we performed double labeling with a neuronal marker, β tubulin III. Y_1_R expression was observed in β tubulin III-positive OSNs (Fig. 3C). However, not all the β tubulin III-positive OSNs were positive for Y_1_R, further suggesting an heterogeneity of the expression in the neuronal population. Therefore, we decided to quantify and compare the relative expression of Y_1_R, using a western blot analysis of the endoturbinates II_b_ and III dissected from the fed and fasted rat hemi-heads contralateral to those used for EOGs.

### Y_1_R expression is upregulated in the OM of fasted rats

OM tissue from the II_b_ and III endoturbinates of fed (n = 5) and fasted (n = 11) rats were dissected from hemi-heads opposite to the EOG-recorded ones. The Y_1_R protein expression level in the OM extracts was quantified on duplicated western blots. Two main bands of the same intensity, of roughly 55 and 70 kDa, were recognized by the Y_1_R antibody on the western blot membranes (Fig. 4A). The application of the pre-absorbed antibody in the same conditions led to the distinct decrease of both the 55 kDa and the 70 kDa band (Fig. S1), thus indicating that the Y_1_R is expressed as two different isoforms in the OM. The expression of these two isoforms was analyzed in fed and fasted animals. Both of them showed an enhanced expression in overnight fasted rat OM, which was significant for the 70 kDa band (+65%) (Fig. 4B).

## Discussion

The present study provides the first demonstration of the NPY involvement in the acute modulation of OSN responses to odorants in rats. Furthermore, we show that this modulation depends on the nutritional status, which regulates the Y_1_R expression in the OM.

Numerous studies have shown the presence of NPY in both the developing and the adult olfactory mucosa of rodents. All these observations clearly showed that NPY plays an important role in the neonatal proliferation of olfactory neuronal progenitor cells [37], [44] and the adult permanent renewal of the OSNs from basal cells [39], [41]. By essence, all of these actions are long-term processes, and relate to the trophic and proliferative role of NPY [38] also described in the hippocampus [54], [55]. In contrast, we show for the first time in mammals that NPY acutely modulates odorant-induced OSNs responses in the OM of adult rats. Interestingly, a similar effect has been characterized in the olfactory epithelium of hungry axolotls [45], suggesting that NPY may play a neuromodulatory role in the peripheral olfactory systems of vertebrates, from amphibians to rodents. It is worth to note that, in both cases NPY transiently increases EOG responses by ∼30%, but solely in hungry animals. Finally, the invertebrate NPY-like peptides, neuropeptides F, have been recently proposed to play a similar modulation of the chemosensory neurons of antennae and maxillary palps of Drosophila [56], highlighting a neuromodulatory mechanism which may be common to mammals and insects.

Our results clearly show that food deprivation up-regulates the responsivity of the rat OM neurons to one of the most potent orexigenic peptide, NPY. Moreover, NPY altered the amplitudes but not the kinetics of EOG responses, and it is generally proposed that EOG amplitudes reflect the number of responding neurons and/or the efficiency of the signal transduction in the OSNs [48]. Since we delivered similar exogenous NPY application to all the tested endoturbinates, the observed hunger-based differences in responses are likely caused by differences in the expression or activity of NPY receptors or other elements of the NPY signal transduction pathway in OSNs, as it was also suggested in hungry axolotls [45].

Using selective NPY receptor agonists, we identified the Y_1_R as the main target of this modulation. Interestingly, most of the neuroproliferative effects of NPY in the OM were also ascribed to the activation of Y_1_R, based on pharmacological results [37], [39], [40], [57]. Whereas several local sources of NPY were identified in the OM, including basal cells [37], OSNs [40], sustentacular cells [40], [41], olfactory ensheathing cells [40], [43] and microvillar cells [42], the distribution of Y_1_R in OM cells was less documented. Using pharmacology and fluorescently-tagged NPY binding approaches, Hansel and coll. (2001a) showed that Y_1_R are expressed in basal progenitor cells. Accordingly, Y_1_R knockout mice show a significant reduction in olfactory neuronal precursor proliferation, which was hypothesized to result in a moderate impairment of olfactory function [40]. Here, using selective antibodies, we show that Y_1_R are also expressed in OSNs of the adult rat OM. Taken together, this localization and the acute effect of exogenously applied NPY on EOG recordings strongly suggest a direct modulatory effect of NPY on Y_1_R expressing OSNs.

The Y_1_R immunostaining in the OM is supported by western analysis of the protein content of this tissue. Two main bands of 55 and 70 kDa were observed for the Y_1_R in the membrane-enriched protein preparations from OM endoturbinates of the recorded animals. The presence of multiple bands for the Y_1_R, has been reported previously [58] and suggested to be a product of post-translational processing or glycosylation of the N-terminus of Y_1_R. The 70 kDa isoform has been repeatedly described as the major glycosylated form of the Y_1_R in neuronal cells [58], [59], [60]. In the present study, a moderate (overnight) fasting induced a significant up-regulation of the 70 kDa Y1R protein expression in the OM. The modulation of Y_1_R level by the nutritional status has been already observed. Indeed, the Y_1_R signaling plays the most prominent role in the stimulation of feeding and obesity, particularly through profound gene expression changes induced by positive or negative states of energy balance in specific regions of the hypothalamus [61]. Noteworthy, moderate food restriction or short-term fasting (overnight) induces an increased expression of Y1R mRNA in the hypothalamus [62], [63]. The increase in Y_1_R protein level in fasted animals OM could be partly responsible for the increased responsiveness of OSNs to exogenously applied NPY observed in EOG. Moreover, the upregulation of NPY responses in the OM of fasted animals may also result from a better signaling efficacy [64] or from a modified balance between desensitization and resensitization of NPY-Y1Rs [65], [66]. Future studies are clearly required to unravel the modulatory mechanisms underlying the action of this peptide in the OM.

Behavioral studies previously demonstrated that fasting increases the olfactory system responsiveness in rats [3], [6]. These reports and other concordant studies repeatedly suggested that fasting affects odors perception mostly through a modulation of the sensitivity of OB neurons to food intake peptides, like orexins and leptin [5], [13], [67]. Similarly, a recent study also suggested that the appetite-stimulating hormone, ghrelin, enhances olfactory sensitivity and exploratory sniffing in rodents and humans [68]. The demonstration that NPY acts as a neuromodulator in the OM adds to the recently growing body of evidences showing that the responsivity to odor of olfactory sensory neurons is also regulated by different food intake modulators, including leptin [15] and insulin [14], [15] in rodents, NPY [45] and endocannabinoids in amphibians [69], [70]. Whereas leptin and insulin, both anorexigenic factors, inhibit the OSN responses to odorants in well-fed animals, oppositely endocannabinoids and NPY, both orexigenic systems, increase the OSN responsivity. Noteworthy, endocannabinoids and NPY effects were respectively up-regulated or observed solely in hungry animals [45], [70] (and present report). Nevertheless, it clearly appears that different food intake regulators oppositely modulate the sense of smell to match the satiety status. Interestingly, some of these food intake modulators similarly regulate the taste signals: leptin decreases, whereas endocannabinoids increase sweet taste sensitivity of taste cells [71], [72]. Taken together, all these concordant data suggest that the sensory nervous system is definitively a target of the nutritional status gauges, and thereby a significant part of the multimodal system of food seeking and food intake regulation.

## Supporting Information

Figure S1 **Immunoblotting for Y_1_R (1∶500, rabbit polyclonal Y1R antibody, ab 73897, Abcam, Cambridge, UK) in OM extracts from fed and fasted rats reveals two main bands (55 and 70 kDa) on the western blot membranes (upper blot).** The use of this Y_1_R antibody pre-adsorbed with the synthetic immunizing peptide (ab82262; Abcam, Cambridge, UK), at a concentration ratio of 1∶10 (antibody:peptide), on the same OM extracts, in the same conditions, led to the distinct decrease of both bands (lower blot).
(TIF)
