# Supplementary figures and images for: Neuropeptide Y Enhances Olfactory Mucosa Responses to Odorant in Hungry Rats
Source: PLoS One. 2012 Sep 14;7(9):e45266. doi: 10.1371/journal.pone.0045266 (PMC3443224; doi:10.1371/journal.pone.0045266)

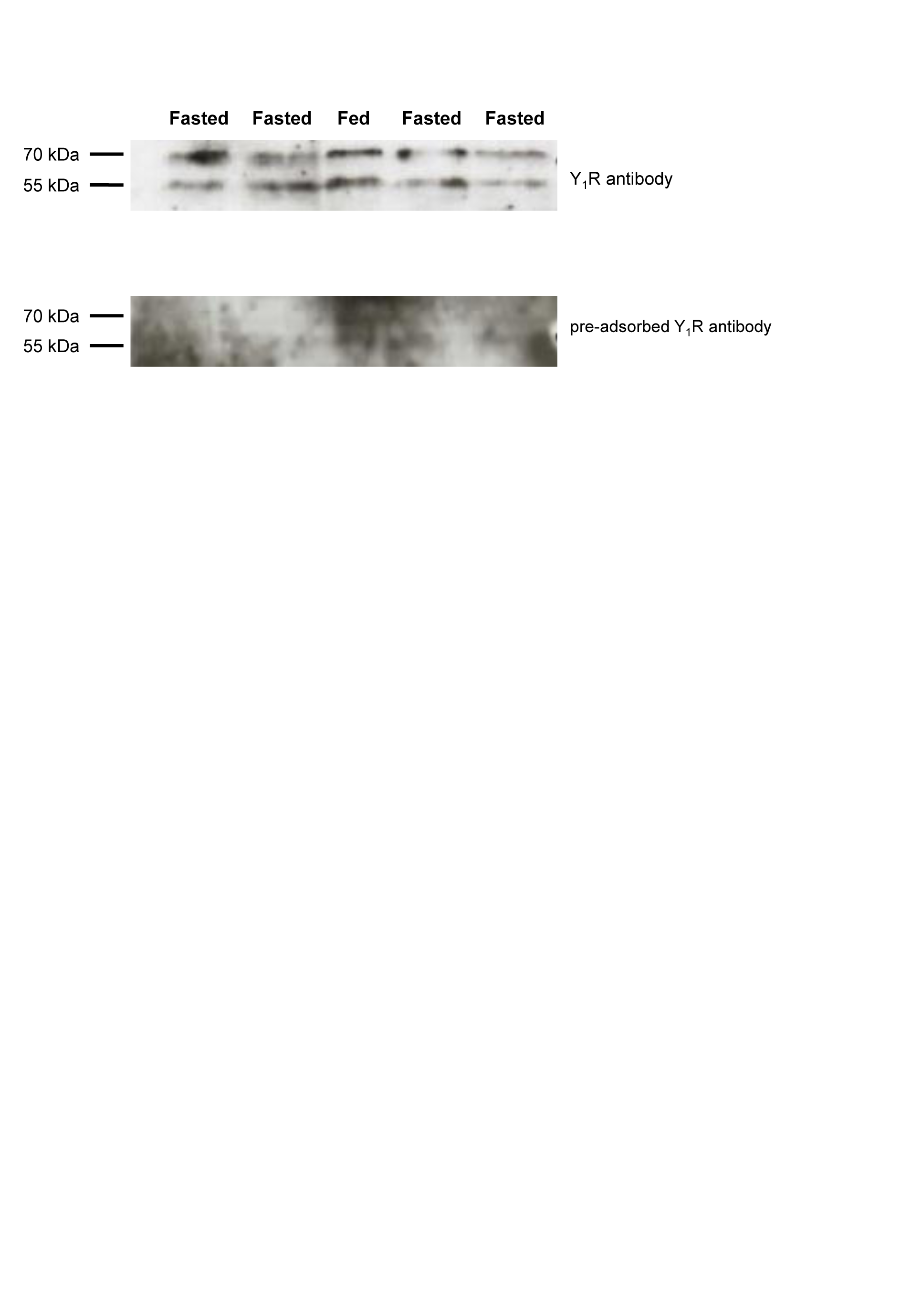

Supplement: Figure S1 — Immunoblotting for Y1R (1∶500, rabbit polyclonal Y1R antibody, ab 73897, Abcam, Cambridge, UK) in OM extracts from fed and fasted rats reveals two main bands (55 and 70 kDa) on the western blot membranes (upper blot). The use of this Y1R antibody pre-adsorbed with the synthetic immunizing peptide (ab82262; Abcam, Cambridge, UK), at a concentration ratio of 1∶10 (antibody:peptide), on the same OM extracts, in the same conditions, led to the distinct decrease of both bands (lower blot). (TIF) [file pone.0045266.s001.tif]
